# Scale development on consumer behavior toward counterfeit drugs in a developing country: a quantitative study exploiting the tools of an evolving paradigm

**DOI:** 10.1186/1471-2458-13-829

**Published:** 2013-09-11

**Authors:** Abubakr A Alfadl, Mohamed Izham b Mohamed Ibrahim, Mohamed Azmi Ahmad Hassali

**Affiliations:** 1 National Drug Quality Control Laboratory, National Medicines and Poisons Board, Federal Ministry of Health, Qasr Street, Khartoum, Sudan; 2 College of Pharmacy, Qatar University, P.O. Box 2713, Doha, Qatar; 3 Discipline of Social and Administrative Pharmacy, School of Pharmaceutical Sciences, Universiti Sains Malaysia, 11800, Penang, Malaysia

**Keywords:** Counterfeit drugs, Behavior, Scale, Developing country, Arabic-speaking Sudanese population

## Abstract

**Background:**

Although desperate need and drug counterfeiting are linked in developing countries, little research has been carried out to address this link, and there is a lack of proper tools and methodology. This study addresses the need for a new methodological approach by developing a scale to aid in understanding the demand side of drug counterfeiting in a developing country.

**Methods:**

The study presents a quantitative, non-representative survey conducted in Sudan. A face-to-face structured interview survey methodology was employed to collect the data from the general population (people in the street) in two phases: pilot (n = 100) and final survey (n = 1003). Data were analyzed by examining means, variances, squared multiple correlations, item-to-total correlations, and the results of an exploratory factor analysis and a confirmatory factor analysis.

**Results:**

As an approach to scale purification, internal consistency was examined and improved. The scale was reduced from 44 to 41 items and Cronbach’s alpha improved from 0.818 to 0.862. Finally, scale items were assessed. The result was an eleven-factor solution. Convergent and discriminant validity were demonstrated.

**Conclusion:**

The results of this study indicate that the “Consumer Behavior Toward Counterfeit Drugs Scale” is a valid, reliable measure with a solid theoretical base. Ultimately, the study offers public health policymakers a valid measurement tool and, consequently, a new methodological approach with which to build a better understanding of the demand side of counterfeit drugs and to develop more effective strategies to combat the problem.

## Background

In recent decades, the counterfeit drugs trade has developed into a substantial threat to both public health and the pharmaceutical industry [1]. According to the World Health Organization (WHO), 60% of counterfeit drug cases occur in less-developed countries where, it is estimated, more than 25% of the drug supply is counterfeit [2]. To combat this problem effectively, it is important to recognize the differences between drug counterfeiting incentives in developing countries and in the developed world, because each requires appropriate and different solutions. In developed countries where medicines are highly accessible, consumers balance financial cost against perceived benefits in all purchases [3]. By contrast, in developing countries, where there is low access to medicines, high drug prices and counterfeiting are linked [4]. According to the WHO, an estimated one-third of the world’s population still lacks regular access to essential drugs, with this figure rising to over 50% in the poorest parts of Africa and Asia [5]. This is reflected in the fact that essential and life-saving drugs, such as antibiotic and antimalarial drugs, are the most affected by counterfeiting [6,7]. Quite often, the quality of these counterfeits is poor, but for life-saving drugs that are not available or not affordable through the regular distribution channels, there is a desperate need-driven demand, allowing counterfeit drugs to be successfully distributed in the market despite their poor quality. Therefore, in developing countries, in order to develop more effective and successful strategies to combat counterfeit drugs, it may be advantageous to include measures targeting the demand side of the problem: this paper addresses the need for a scale to aid in understanding the demand for counterfeit drugs.

Even though the counterfeiting of products compromises quality, consumers may be willing to overlook this fact because of attitudinal and/or motivational factors. Therefore, governments and businesses must emphasize the need for more research to determine how to target these consumers and what manner of appeal to use in order to eliminate the demand for counterfeit drugs [3]. Unfortunately, while consumer research has extensively examined consumer behavior toward counterfeit luxury goods or software piracy (see, for example, Albers-Miller [8]), consumer behavior toward counterfeit drugs (CBTCD) has been largely ignored [1]. Consequently, the constructs most closely related logically to CBTCD and the measures available are those measuring consumer behavior toward counterfeit luxury goods. Despite the apparent similarities of these constructs and, in some cases, their theoretical relationship to the counterfeiting of medicines, none of these constructs captures the construct of CBTCD. Moreover, findings from previous research have suggested that consumer behavior toward counterfeits tends to be product specific [9-11]. Addressing this gap in the theoretical literature by offering a methodological contribution—that is, a valid and reliable measure of CBTCD—would allow public health policymakers and marketing managers to understand the demand side of the problem of counterfeit drugs better. This research was carried out as a step in that direction, using the Theory of Planned Behavior (TPB) [12] as a framework model. The TPB was originally designed to predict people’s intention to buy as a secure indicator of actual purchase. According to the TPB, people act in accordance with their intentions and perceptions of control over the behavior, while intentions are influenced by attitudes toward the behavior, subjective norms, and perceptions of behavioral control [12,13].

It should also be noted that most of the studies on consumers’ perceptions of counterfeiting have focused on consumers in the developed world and little is known about those in developing countries. If public health policymakers or marketing managers wish to measure the extent to which consumers in the developing world have a high or low tendency to acquire counterfeit drugs, they have no valid and reliable instrument to measure the trait. Moreover, an empirical study regarding the basic characteristics of consumers vulnerable to counterfeit drugs could provide an anchor for further methodological investigations into this under-studied area. This research aims to contribute to filling that gap by developing a measurement scale. Additional file 1 was developed to analyze the factors that may influence consumers in Sudan to purchase counterfeit drugs. In other words, it aims to develop a sound multidimensional measure of CBTCD in a developing country setting based on an accepted practice while exploiting the tools of an evolving paradigm.

## Methods

### Scale development

**Figure 1:**
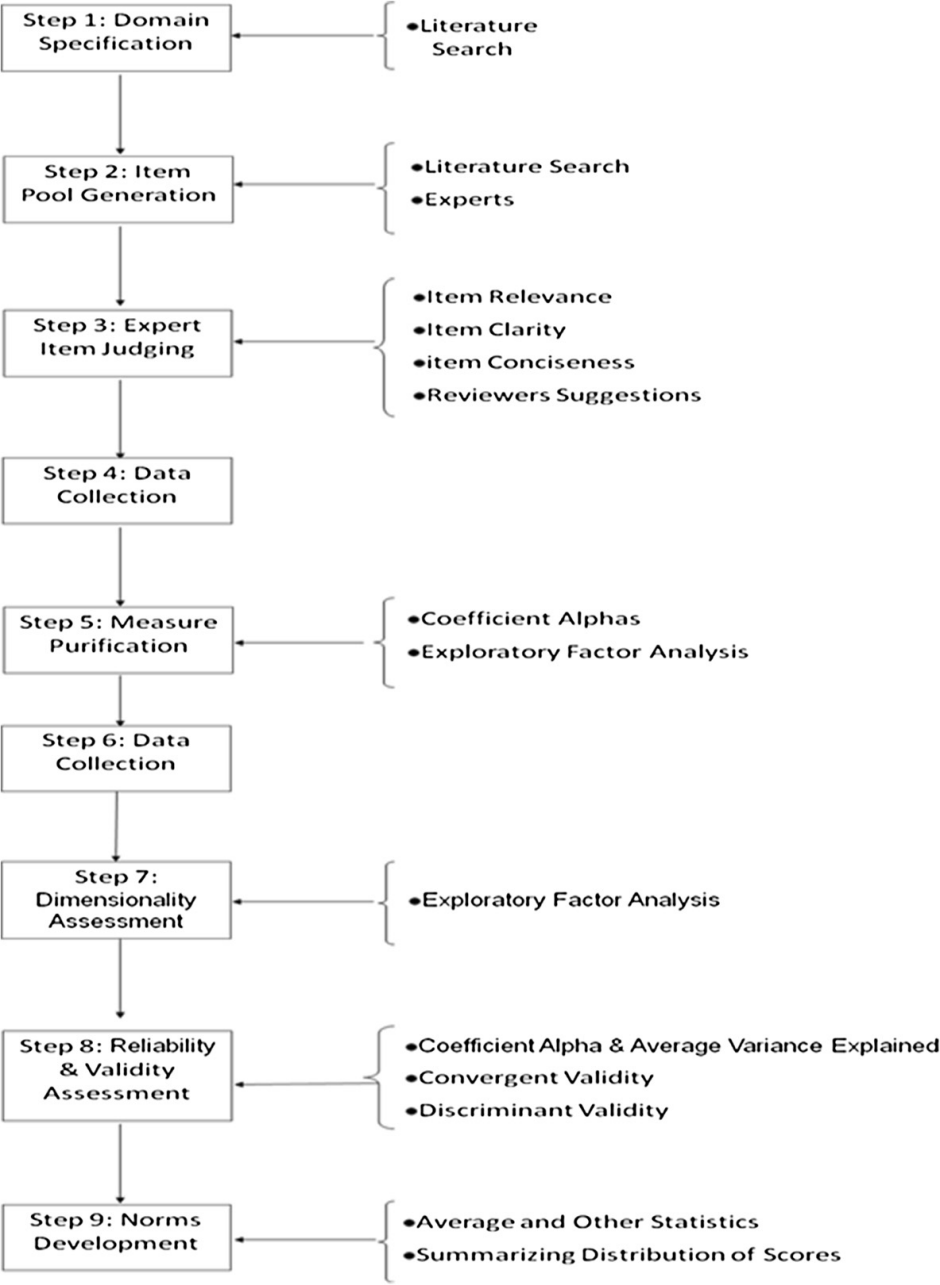
Scale development procedure.

In key respects, scale construction followed the approaches developed by Churchill [14] and DeVellis [15] (Figure 1).

### Specifying the domain of the constructs

We initially worked to specify the domain of the constructs because this is regarded as the first step in developing new measures [14]. A slight modification was made to the model proposed by the TPB [13] by naming the perceived behavioral control component “motivation.” This was to allow for the dimension of need [16] in purchasing counterfeit drugs as the study was conducted in a resource-limited setting. Nevertheless, both perceived behavioral control and motivation components are concerned with the availability of resources (e.g., money, time, etc.) [13,16]. It was anticipated that the CBTCD scale would have four main dimensions, namely: attitude, subjective norms, motivation, and behavioral intention. Thus, in this study, the dimensions of the scale were hypothesized as follows: beliefs about counterfeit drugs will influence attitude and subjective norms, while non-availability of resources will influence the motivation to purchase. Attitude, subjective norms, and motivation will influence the intention to purchase counterfeit drugs, and, consequently, intention will mediate and predict counterfeit drugs purchasing behavior [12,13,16].

### Item generation

The first step in scale development, after specifying the domain, was to develop item sets for each of the four main dimensions. Efforts were directed, in particular, to creating a scale that had (1) broad domain coverage in support of content validity, (2) adequate internal consistency reliability, and (3) convergent and discriminant validity. Based on an extensive literature review of the counterfeiting of products and consumer behavior, supplemented by 11 in-depth interviews with health policymakers and community pharmacists [17], an initial pool of 69 variables reflecting different aspects of consumers’ beliefs about and motivation to purchase counterfeit drugs was generated.

These items were submitted to a panel of expert judges (public health and marketing professionals), together with the definitions of all the dimensions of the scale. The experts were asked to rate each of the 69 items in the initial pool using a three-point scale (1 = not representative, 2 = somewhat representative, 3 = clearly representative) to indicate the extent to which it represented the specific dimension. To assess face and content validity, a decision rule that focused on the overall evaluation of all the judges was used [18,19]; that is, items rated by at least 80% of the judges as at least somewhat representative, or by 60% as clearly representative, were retained. These items were further refined using the paradigm of scale development developed by Churchill [14] and DeVellis [15].

### Study 1: scale refinement and purification

#### Sample and procedure

Following the procedure set by Churchill [14], early data collection for item refinement was undertaken. Before starting data collection, formal ethical approval was granted from the Federal Ministry of Health–Research Directorate. All respondents verbally consented to be interviewed. A small number of respondents (n = 100) were targeted, distinct from those interviewed in the final survey but recruited in exactly the same way and with characteristics similar to those approached in the actual final survey [20].

#### Factor and item analysis

The CBTCD scale items were factor analyzed in order to enhance understanding of the measurement quality and to determine and simplify the factor structure for the observed variables based on the correlation matrix. The resulting eigenvalues, scree test plot, and explained variance were employed as decision criteria to determine the number of factors to retain. The main concern was the reduction of the data set [21], however. Items were retained if (1) they loaded 0.4 or more on a factor, (2) did not load more than 0.4 on two factors, and (3) the reliability analysis indicated an item-total correlation of more than 0.4 [22].

### Study 2: scale validation

A second round of data collection was carried out in order to assess further the factor structure (unidimensionality) and reliability of the purified scale of 41 items, as well as to establish the convergent and discriminant validity of all factors in the scale.

#### Sample and procedure

A convenience sample of Sudanese consumers was used, and 1003 questionnaires were completed using a face-to-face survey procedure. The target population comprised people living in the states of Khartoum and Gadaref. The sampling approach was based on the availability of participants (convenient sample). Although availability sampling is a non-probability technique, the authors nonetheless attempted to make the sample representative. This representativeness was supported by using the available demographic and socio-economic data to predict the participant population required. This then played a role in the sampling technique that was used for this study. The initial availability sampling relied on participants who agreed to participate. As the study progressed, data collectors changed to a more purposive approach to sampling. This meant that it was possible to ask at the participant-selection stage for more participants with a particular characteristic, for example, requesting a wider sample of females or from a particular age group. However, since this study was exploratory in nature, being the first attempt to develop a CBTCD scale, a convenience sample can be considered valid [23]. The eligibility criteria were that participants were Sudanese, willing to participate, and aged above 18 years. Those who did not know Arabic were excluded from the study.

A face-to-face interview technique was used as the method for data collection to maximize the response rate and to give the data collectors the opportunity to explain the questions to the respondents. Data collectors were provided with a brief description of the construct dimensions, and all items in the scale were clearly explained to them to ensure consistency. It was accepted that the interviewers might shorten the question, simplify the language, or suggest answers by changing how questions were worded. To account for this and ensure that all interviewees received the same set of questions asked in the same order, the interviewers were asked to do every interview in an identical manner. Very little flexibility was allowed them in helping respondents to understand the questions.

#### Second sample analysis

This second sample was used to test the unidimensionality and validity of the scale. Inter-correlations among variables were tested, and after obtaining good results, Exploratory Factor Analysis (EFA) was performed employing Statistical Package for Social Sciences (SPSS) version 16 to assess unidimensionality [22,24]. Although confirmatory factor analysis (CFA) is considered a more powerful and more flexible technique than EFA for such an assessment, EFA was used because it has been widely suggested as the appropriate tool when a new scale is being developed [22,24]. To assess validity, CFA was performed employing AMOS graphics version 5.0 [25].

## Results

### Expert judgment

Expert judgment resulted in the original edited item pool being reduced from 69 items to 47 items. Three additional items were removed on the specific written recommendation of expert judges. The application of this judging procedure reduced the number of items across the four dimensions (attitude, subjective norms, motivation, and behavioral intention) to 29, 11, 2, and 2, respectively.

### Study 1: scale refinement and purification

#### Sample and procedure

The participants were relatively young, with 37% between 18 and 27 years of age. Respondents’ ages ranged from 18 to 75 years old. The sample was well educated: 38% of the respondents were university or college graduates, and only 5% had a level of education lower than primary school. As for occupation, up to 52% of the respondents were unemployed (homemakers, students, or jobless) (Tables 1 and 2).

**Table 1:** Demographic profile of the first and second samples

**Variable**	**Category**	**Frequency %**	
		**1st sample**	**2nd sample**
**Gender**	Female	48.0	47.1
	Male	52.0	52.9
**Education**	Less than primary	5.0	7.3
	Primary	19.0	15.7
	Secondary	38.0	36.6
	Graduate	38.0	38.5
**Working status**	Unemployed	32.5	50.4
	Skilled day labor	1.3	6.5
	Unskilled day labor	14.4	5.7
	Professional governmental employer	13.1	18.4
	Non-professional governmental employer	4.4	4.3
	Professional non-governmental employer	8.1	6.6
	Non-professional non-governmental employer	7.5	3.1
	Professional businessman	3.1	1.5
	Non-professional businessman	9.4	1.5
	Retired	6.3	2.0

**Table 2:** Demographic profile of the first and second samples

**Variable**	**Mean**		**Median**		**Standard deviation**	
	**1st sample**	**2nd sample**	**1st sample**	**2nd sample**	**1st sample**	**2nd sample**
**Age**	30.2	32.7	30.0	30.0	6.5	11.8
**Annual income**	3440.5	3601.5	2720.0	2800.0	2320.4	2808.6

#### Factor and item analysis

Means, variances, standard deviations, and alpha coefficients are presented in Table 3. The eigenvalue criterion retains only those factors having eigenvalues greater than one (Table 4), and the number of factors retained using the scree test criterion is based upon the point at which the curve begins to straighten. The results of the initial EFA using a varimax rotation yielded 13 factors that explained 72.2% of the variance in the data. Items with a low item-total correlation (< 0.40), low factor loadings (< 0.40), or significant cross-factor loadings (> 0.40) were omitted [26]. The remaining 41 items loaded on 11 factors, explaining 62.2% of the variance in the data, thus supporting the multidimensional conceptualization of the CBTCD scale. Each factor consists of two to nine items that seem to be reliable measures (sub-scales), with Cronbach’s alpha values of more than 0.50, with the exception of accessibility (α = 0.44). The average scores for each sub-scale were also normally distributed and showed adequate variance (Table 3).

**Table 3:** Scale and reliability statistics (First sample)

	**Mean**	**Variance**	**Std. deviation**	**Cronbach’s Alpha**	**N of items**
Perceived product attributes (PA)	38.92	23.437	4.841	0.566	10
Perceived risks (PR)	21.11	11.432	3.381	0.749	5
Risk averseness (RA)	17.90	4.798	2.190	0.567	4
Price quality inference (PQ)	14.63	16.619	4.077	0.788	4
Awareness of societal consequences (ASC)	12.22	6.396	2.529	0.685	3
Subjective norms (SN)	7.56	4.027	2.007	0.804	2
Affordability-related perceptions (AF)	15.35	19.806	4.450	0.691	5
Availability-related perceptions (AV)	9.42	8.529	2.920	0.579	3
Accessibility-related perceptions (AC)	9.53	7.605	2.758	0.437	3
Behavioral intention (BI)	7.10	4.010	2.003	0.520	2
Total Scale	153.56	340.723	18.459	0.862	41

**Table 4:** Eigenvalues and total variance explained

**Component**	**Initial eigenvalues**			**Rotation sums of squared loadings**		
	**Total**	**% of variance**	**Cumulative %**	**Total**	**% of variance**	**Cumulative %**
1	7.610	18.560	18.560	4.824	11.765	11.765
2	4.263	10.399	28.959	2.926	7.137	18.902
3	2.341	5.710	34.668	2.757	6.724	25.626
4	2.291	5.589	40.257	2.588	6.312	31.938
5	2.073	5.056	45.313	2.363	5.764	37.701
6	1.829	4.461	49.775	2.219	5.413	43.114
7	1.660	4.048	53.823	2.052	5.004	48.118
8	1.521	3.709	57.532	1.939	4.729	52.847
9	1.405	3.428	60.960	1.781	4.343	57.190
10	1.383	3.373	64.334	1.734	4.229	61.419
11	1.167	2.846	67.180	1.634	3.987	65.405
12	1.047	2.554	69.734	1.562	3.809	69.215
13	1.017	2.480	72.215	1.230	3.000	72.215

### Study 2: scale validation

#### Sample and procedure

The sample consisted of almost balanced proportions of male (47.1%) and female respondents (52.9%). Although the sample was young (mean age = 32.7 years), a wide range of ages was represented (minimum = 18 years old, maximum = 75 years old). All education levels were represented: 7.3% less than primary school; 15.7% primary; 36.6% secondary; and 38.5% university or college. Most of the respondents were either unemployed or non-governmental employers. For annual income, a wide range was reported (USD30–28800) with a median of about USD 2800 (Tables 1 and 2).

#### Unidimensionality assessment

After inter-correlations among variables were tested and good results for Bartlett’s test of sphericity (< 0.05) and the Kaiser-Meyer-Olkin measure of sampling adequacy (KMO) (> 0.50) [26] were obtained, EFA was performed. The authors were concerned primarily with determining the minimum number of factors needed to account for the maximum portion of the variance represented in the original set of variables. That is to say, the main concern was to reduce the dataset by expressing a large number of indicators by means of a smaller set of linear composites, commonly known as factors. Therefore, component analysis was selected. In addition, because the objective of the authors was to utilize the factor results in a subsequent statistical analysis, they selected an orthogonal rotation procedure. A varimax method was used because it has proved to be very successful as an analytic approach to obtaining an orthogonal rotation factor. Items with factor loadings less than 0.3 or with cross-loading above 0.4 were deleted. All remaining items loaded significantly (> 0.50) on their constructs, explaining more than 50% of variance [24] of each respective construct (Table 5). This demonstrates that every set of items empirically measured a single dimension.

**Table 5:** Factor structure (Second sample)

**Factors/items**	**Factor loading**	**α**	**AVE**	**KMO**	**Bartlett’s**
**Perceived product attributes (PA)**		**0.66**	**53.6%**	**0.86**	**p < 0.001**
PA1. Authentic better quality than non-authentic	0.57				
PA2. Non-authentic as good as authentic	−0.53				
PA3. Purchasing non-authentic drugs worthless	0.73				
PA4. Non-authentic drugs not worth buying	0.73				
PA5. Authentic drugs more reliable than non-authentic	0.74				
PA6. Authentic drugs perform much better than non-authentic	0.74				
PA7. Authentic drugs worth the money they cost	0.72				
PA8. Cognitive belief regarding attribute of non-authentic drugs negative	0.68				
PA9. Thoughts and feelings towards non-authentic drugs ambivalent	0.85				
**Perceived risk (PR)**		**0.89**	**70.4%**	**0.87**	**p < 0.001**
PR1. Risk when buying non-authentic drugs high	0.81				
PR2. Probability that non-authentic drugs don’t work high	0.84				
PR3. Spending money on non-authentic drugs bad decision	0.85				
PR4. Non-authentic drugs very dangerous	0.88				
PR5. Purchasing non-authentic drugs risky	0.82				
**Risk averseness (RA)**		**0.75**	**57.9%**	**0.77**	**p < 0.001**
RA1. When buying prefer not taking risk	0.76				
RA2. Like to be sure product good before buying	0.77				
RA3. Don’t like to feel uncertainty when buying something	0.77				
RA4. Always avoid risky things	0.74				
**Price quality inference (PQ)**		**0.80**	**63.7%**	**0.75**	**p < 0.001**
PQ1. Higher price, higher quality	0.89				
PQ2. Have to pay more for best	0.91				
PQ3. Price premium of authentic drug compared to non-authentic justified	0.43				
PQ4. Price good indicator of quality	0.86				
**Awareness of societal consequences (ASC)**		**0.65**	**61.1%**	**0.59**	**p < 0.001**
ASC1. Purchasing non-authentic drugs harm national economy	0.86				
ASC2. Purchasing non-authentic drugs undermine national health system	0.86				
ASC3. Purchasing non-authentic drugs discourage manufacturers of legitimate drugs	0.59				
**Subjective norms (SN)**		**0.67**	**75.4%**	**0.50**	**p < 0.001**
SN1. Relatives and friends approve decision to buy non-authentic drugs	0.87				
SN2. Relatives and friends think I should buy non-authentic drugs	0.87				
**Affordability-related perceptions (AF)**		**0.87**	**72.2%**	**0.83**	**p < 0.001**
AF1. Buying non-authentic drugs because authentic unaffordable	0.81				
AF2. Buying non-authentic drugs because affordable	0.89				
AF3. Buying non-authentic drugs because not ready to pay price of authentic	0.87				
AF4. Unaffordable prices of authentic drugs cause of buying non-authentic	0.84				
**Availability-related perceptions (AV)**		**0.75**	**80.2%**	**0.50**	**p < 0.001**
AV1. Buying non-authentic drugs because authentic not available	0.90				
AV2. Non-availability of authentic drugs cause buying of non-authentic	0.90				
**Accessibility-related perceptions (AC)**		**0.81**	**84.1%**	**0.50**	**p < 0.001**
AC1. Buying non-authentic drugs because authentic not accessible	0.92				
AC2. Non-accessibility of authentic drugs cause of buying non-authentic	0.92				
**Behavioral intention (BI)**		**0.65**	**74.3%**	**0.50**	**p < 0.001**
BI1. May buy non-authentic drugs in future	0.86				
BI2. There are favorable things to be said about non-authentic drugs	0.86				

Extraction Method: Principal Component Analysis. Rotation Method: Varimax with Kaiser Normalization.

#### Reliability assessment

A reliability assessment of the 10 constructs revealed that the Cronbach’s alpha of internal consistency ranged from 0.65 to 0.89, above the benchmark of 0.60, and that the average variance extracted (AVE) ranged from 53.6% to 84.1%, also above the benchmark of 50% [24] (Table 5). These results indicate the strong reliability of the scale.

### Validity

#### Convergent validity

The convergent validity of the scale was tested by evaluating the AVE and reliability estimates (Cronbach’s alpha). As shown in Table 5, the AVE of all constructs exceeded 0.50, while all reliability estimates were well above 0.60 [24]. Thus, convergent validity was demonstrated.

#### Discriminant validity

Discriminant validity was tested on the basis of the Fornell and Larcker approach [25]. This approach states that the square root of the AVE for each construct should exceed the correlation estimate between any two constructs. The results obtained through CFA (see [27]) indicate that all the constructs achieve this criterion as none of the off-diagonal elements exceeded the respective diagonal element.

## Discussion

The vast majority of previous research on drug counterfeiting has been limited in scope in that it focused on the supply side of the problem. One objective of this study was to begin to fill this gap in the literature. To this end, based on accepted methods of scale construction, this study has developed a scale to measure CBTCD in a developing country setting. After a thorough examination of the literature on product counterfeiting and consumer behavior, and based on TPB, it was assumed that consumers in Sudan may sometimes intentionally buy counterfeit drugs. Four main influencing factors were identified, namely, attitude, subjective norms, motivation, and behavioral intention. A qualitative study conducted with health policymakers and community pharmacists in two state offered some empirical support for the relevance of these factors [17]. Prospective items for use in the CBTCD scale were aggregated. The result was a pool of 69 prospective items. Seven expert judges in the areas of public health and marketing evaluated the items for face and content validity. The process produced 44 items that were judged as having both face validity and content validity. The 44 items produced from the item generation and expert judging were tested on a sample of 100 Sudanese consumers, and a principal component analysis was performed on the data. An iterative purification process produced a preliminary 11-factor structure for the CBTCD scale. These factors exhibited acceptable preliminary reliability (coefficient alpha). Item-total correlations and inter-item correlations both met recommended thresholds. Some researchers have suggested that maximizing internal consistency is a misguided approach to scale development, however; particularly because items with the highest item-total correlations often share a considerable proportion of variance so subtests containing only these items will probably not maximize variance in the original test score. Moreover, by maximizing reliability, researchers may have created a narrower measure with suppressed validity coefficients. Therefore, statistical thresholds for item retention/deletion are assumed merely to be guidelines and should not result in the deletion of items that have face and/or content validity; rather, they should be retained for the next round of the study [28]. For this reason, score-reduction decisions in the current research were made with great circumspection, and some items were retained in the scale for the next test (EFA) despite their low item-total correlations and SMCs.

The psychometric properties of the scale were assessed with a second sample of Sudanese adult consumers, and a 10-dimensional scale of 38 items was developed covering the four factors proposed by TPB (for detailed dispersion of dimensions through the factors and items through the dimensions, refer to the scale). The results showed that the items converged toward their corresponding factors and that the 10 sub-scales composing the instrument were reliable.

Prior to this research, no scale existed to measure CBTCD, which prevented empirical examination of the construct and its relationship with other consumer behavior constructs. The creation of a CBTCD measure with acceptable psychometric properties provides public health policymakers with an opportunity to examine empirically this important consumer trait. More specifically, the CBTCD scale developed and validated in this study may help researchers and public health policymakers to determine empirically the extent to which consumers favor counterfeit drugs over authentic ones under the influence of certain factors and, moreover, to identify the variables that may drive the intention to purchase counterfeit drugs. This knowledge may in turn be used to develop strategies that are more efficient in combating the demand side of the drug counterfeiting problem.

Furthermore, the theoretical development of this construct, along with the construction of an empirical measure, will allow public health policymakers and marketing managers interested in dissuading consumers from opting for counterfeit drugs to begin to understand an important consumer trait. Without the ability to understand fully the nature of individuals with this trait, it would be difficult to appeal to consumers who exhibit the tendency to purchase counterfeit drugs. Knowing what type of factors significantly affect consumers’ attitudes and motivate them to behave in a manner that favors the purchase of counterfeit drugs is the first step in enabling public health policymakers and marketing practitioners to refine their policy, marketing, and advertising strategies in order to persuade these consumers to change their behavior. With the help of the CBTCD scale, it will be possible to estimate the relative importance of each influencing factor in predicting the behavioral intention of the consumer and, consequently, to adapt strategies to combat the problem so that they are more effective. In addition, several scholars have called for systematic empirical research on the demand side of counterfeit products in general and drug counterfeiting in particular (e.g., [1,3,29]). The lack of empirical research in this area may have been caused, in part, by the lack of a scale to measure CBTCD. The CBTCD scale should facilitate further empirical studies into the demand side of the drug counterfeiting problem and, thus, a more refined and efficient policy to combat the problem. The CBTCD scale yields a range of scores that can be used to assess the degree of vulnerability to counterfeit drugs. According to the CBTCD scale, a score of 151 to 190 indicates low vulnerability, 81 to 150 indicates moderate vulnerability, and 38 to 80 indicates high vulnerability.

Despite its contribution, this study has limitations that should be addressed in further research. It is evident from the literature that researching the demand side of the counterfeit drug problem is more important in developing countries where high, unaffordable, drug prices and counterfeiting are linked [1]. Accordingly, the development of an instrument for the measurement of CBTCD seems especially relevant in developing countries. Nevertheless, further research is also to be encouraged in countries other than Sudan, because previous research has shown that national culture has an impact on consumer behavior toward counterfeit products [30-32]. In addition, as is generally the case with research dealing with sensitive issues, people try to appear unselfish and more society-oriented than they actually are. There is, therefore, always the possibility of a social desirability bias, which, it could be argued, is inherent in all surveys that concern consumers’ willingness to adopt socially undesirable behaviors [33-35]. Further research involving the CBTCD scale should therefore consider including an independent measure of social desirability, such as that developed by Crowne and Marlowe [36].

## Conclusion

The authors propose that CBTCD be measured by the CBTCD scale developed in the current study. It is a scale of 37 items using a 5-point Likert-type response format. The results indicate that the CBTCD scale is a valid, reliable scale with a solid theoretical base. Marketing managers and public health officers could employ this scale to gain better knowledge about counterfeit drug problems in a specific market and then use this knowledge according to their particular needs.

## Competing interests

The authors declare that they have no conflicts of interest to disclose.

## Authors’ contributions

AAA participated in the design of the study, performed the statistical analysis, and drafted the manuscript. MMI participated in the design of the study and critically reviewed the draft of the manuscript. MAH participated in the design of the study and critically reviewed the draft of the manuscript. All authors read and approved the final manuscript.

## Pre-publication history

The pre-publication history for this paper can be accessed here:

http://www.biomedcentral.com/1471-2458/13/829/prepub

## Supplementary Material

Additional file 1 Scale items. Click here for file
