# Supplementary material for: Scale development on consumer behavior toward counterfeit drugs in a developing country: a quantitative study exploiting the tools of an evolving paradigm
Source: BMC Public Health. 2013 Sep 11;13:829. doi: 10.1186/1471-2458-13-829 (PMC3852329; doi:10.1186/1471-2458-13-829)
Supplement: Additional file 1 — Scale items. [file 1471-2458-13-829-S1.doc]

**SCALE ITEMS**

**Part 1: ATTITUDE**

**a/ Perceived Product Attributes (PA)**: The smaller the differential perceived by consumers between counterfeit and authentic drug on these attributes the higher their purchase intention and vice versa.

1) Authentic drugs are often of better quality than non-authentic drugs.

2) Non-authentic drugs are just as good as authentic drugs.

3) Purchasing non-authentic drugs is worthless.

4) Non-authentic drugs are not worth buying.

5) Authentic drugs are more reliable than non-authentic.

6) Authentic drugs perform much better than non-authentic drugs.

7) Authentic drugs are worth the money they cost.

8) Overall, my cognitive believe regarding the attributes of non-authentic drugs is negative.

9) I would describe my thoughts and feelings towards non-authentic drugs as ambivalent.

**b/ Perceived Risks (PR)**: The magnitude of consequences and the probabilities that these consequences may occur if non-authentic drug is acquired.

1) The risk that I take when I buy non-authentic drug is high.

2) There is high probability that the non-authentic drug doesn’t work.

3) Spending money with non-authentic drug might be a bad decision.

4) Generally speaking, non-authentic drugs can be very dangerous.

5) Purchasing non-authentic drugs is quite risky.

**c/ Risk Averseness (RA)**: The propensity to avoid taking risks.

1) When I buy something, I prefer not taking risks.

2) I like to be sure the product is a good one before buying it.

3) I don’t like to feel uncertainty when I buy something.

4) I always avoid risky things.

**d/ Price-Quality Inference (PQ)**: Consumer tendency to believe that “high (low) price means high (low) quality”

1) Generally speaking, the higher the price of a drug, the higher the quality.

2) You have always to pay a bit more for the best.

3) The price premium of authentic drug compare to non-authentic drug is mostly justified.

4) The price of a drug is a good indicator of its quality.

**e/ Awareness of Societal Consequences (ASQ)**

1) Purchasing non-authentic drugs harm the economy of my country through loss of taxation revenue.

2) Purchasing non-authentic drugs undermining the national health care system of my country.

3) Purchasing non-authentic drugs discourage manufacturers of authentic drugs from investment in research and development and hence slow development of new effective drugs.

**Part 2: SUBJECTIVE NORM**

1) My relatives and friends approve my decision to buy non-authentic drugs.

2) My relatives and friends think that I should buy non-authentic drugs.

**Part 3: MOTIVATION**

**a/ Affordability (AF)**

1) Generally speaking, one of the reasons for buying non-authentic drugs is that the prices of authentic drugs are unaffordable.

2) Generally speaking, one of the reasons for buying non-authentic drugs is that it has affordable prices.

3) One of the reasons for buying non-authentic drugs is that I would not be ready to pay the price of the authentic drugs although I prefer them.

4) Unaffordable prices of authentic drugs may cause me to buy non-authentic drugs.

**b/ Availability (AV)**

1) Generally speaking, one of the reasons for buying non-authentic drugs is that authentic drugs are not always available.

2) Non availability of authentic drugs may cause me to buy non-authentic drugs.

**c/ Accessibility (AC)**

1) Generally speaking, one of the reasons for buying non-authentic drugs is that authentic drugs are not always accessible.

2) For me, purchasing non-authentic drug would not be an option even if the authentic drug is not accessible.

3) Non accessibility of authentic drugs may cause me to buy non-authentic drugs.

**Part 4: BEHAVIOURAL INTENTIONS**

1) It is likely that I may buy non-authentic drug in the future.

2) Still there is a chance that I say favourable things about non-authentic drugs.

**Scale Format**: A five-point Likert scale response format ranging from “1 = strongly disagree” to “5 = strongly agree” was chosen.

| **Total score** | **Level of vulnerability to counterfeit drugs** |
| --- | --- |
| 151 - 190 | Low |
| 81 - 150 | Medium |
| 38 - 80 | High |
